# Separating Recognition Processes of Declarative Memory via Anodal tDCS: Boosting Old Item Recognition by Temporal and New Item Detection by Parietal Stimulation

**DOI:** 10.1371/journal.pone.0123085

**Published:** 2015-03-27

**Authors:** Alberto Pisoni, Zsolt Turi, Almuth Raithel, Géza Gergely Ambrus, Ivan Alekseichuk, Annekathrin Schacht, Walter Paulus, Andrea Antal

**Affiliations:** 1 Department of Clinical Neurophysiology, University Medical Center, Georg-August University, Göttingen, Germany; 2 Department of Psychology, University of Milano-Bicocca, Milano, Italy; 3 Institute of Psychology, Friedrich Schiller University, Jena, Germany; 4 CRC Text Structures, Georg-August University, Göttingen, Germany; Harvard Medical School, UNITED STATES

## Abstract

There is emerging evidence from imaging studies that parietal and temporal cortices act together to achieve successful recognition of declarative information; nevertheless, the precise role of these regions remains elusive. To evaluate the role of these brain areas in declarative memory retrieval, we applied bilateral tDCS, with anode over the left and cathode over the right parietal or temporal cortices separately, during the recognition phase of a verbal learning paradigm using a balanced old-new decision task. In a parallel group design, we tested three different groups of healthy adults, matched for demographic and neurocognitive status: two groups received bilateral active stimulation of either the parietal or the temporal cortex, while a third group received sham stimulation. Accuracy, discriminability index (d’) and reaction times of recognition memory performance were measurements of interest. The d’ sensitivity index and accuracy percentage improved in both active stimulation groups, as compared with the sham one, while reaction times remained unaffected. Moreover, the analysis of accuracy revealed a different effect of tDCS for old and new item recognition. While the temporal group showed enhanced performance for old item recognition, the parietal group was better at correctly recognising new ones. Our results support an active role of both of these areas in memory retrieval, possibly underpinning different stages of the recognition process.

## Introduction

Memory plays an essential role in human cognition and it encompasses several different processes—temporary, dynamic storage and manipulation of information or working memory (WM) [1] as well as long-term memory (LTM) representations [2]. According to a prominent model of human LTM [2], declarative memory is the component of storing factual information, general knowledge, and contextual details of past events; it also serves to organise this information into coherent episodes or facts, which can be consciously accessed later (for a review see: [3]). Multiple brain regions are assumed to interact in order to coordinate these complex processes, both for encoding and retrieval of declarative memories [4–6]. Specifically, it has been proposed that memory formation entails a complex interaction between the hippocampus and a widespread network of cortical regions [7–9], including the temporal, prefrontal and parietal cortices, although the exact mnemonic functions of these areas are still under discussion (for reviews see: [10,11]).

Non-invasive brain stimulation (NiBS) techniques have the potential to further improve our knowledge about the functional and neural correlates of declarative memory by directly manipulating the neural activity of target brain areas. Transcranial direct current stimulation (tDCS), is one of the most extensively used NiBS techniques. During tDCS, a constant, low intensity electrical current is applied to the head [12], which is capable of inducing polarity dependent changes either by de- or hyperpolarising neurons’ resting membrane potentials and causing a reversible increase or decrease in cortical excitability [13]. The relatively long lasting after-effects of tDCS have been demonstrated both at the physiological [12] and behavioural level, encompassing higher order cognitive functions, including LTM [14]. Indeed, previous studies on declarative memory found promising improvements in recognition of encoded material when tDCS was applied in either the learning [15,16] and/or in the recognition phase [14].

Previous studies reported that the activation state of the frontal and parietal regions during memory recognition plays a critical role in recognition success (e.g., [17]). When participants received tDCS over the left and right dorsolateral prefrontal cortices (DLPFC) during the recognition phase of a memory task, a slight improvement was observed following left anodal tDCS (a-tDCS) and a significant decrease following left cathodal tDCS (c-tDCS) [14]. Nevertheless, widespread brain regions have been implicated in encoded material recognition, including not only the frontal but also the temporal and parietal cortices, though this latter area is traditionally linked to attention processes [18].

Although the specific functional contribution of the parietal cortex to human declarative LTM is still debated (for a review see: [19]), it has been proposed that this area could be involved in different stages of memory processing, from encoding to retrieval [20]. In particular, it could act as a mnemonic accumulator, in which a signal-detection process might take place, comparing integrated recollective and familiarity-based memory strength signals with a decision criterion [21]. Alternatively, it might underlie a memory orienting mechanism, focusing attention on internal memory representation linked to recollective oriented constraints, in order to achieve a successful memory performance [22–23], but see [24]. It has also been proposed that the left parietal cortex might perform as an output episodic buffer [25], in which stored information is transferred and activated in order to be processed [26].

In line with these previous findings, a recent study, which applied bilateral parietal stimulation during the encoding phase of a verbal learning task, has found a small improvement in recognition memory performance [15]. Conversely, in a task devised to elicit false memories (e.g. Deese—Roediger—McDermott; DRM paradigms), false recognition rate was increased after bilateral stimulation of the parietal cortex [27]. The beneficial effect of tDCS in episodic LTM retrieval was also observed during temporal lobe stimulation using the same DRM paradigm [28]. Although the false memory rate was significantly reduced under tDCS as compared with the sham condition, the exact effect of stimulation timing remained uncertain, since tDCS was applied both during the encoding as well as the recognition phase of the memory task. Consequently, it is still unclear whether tDCS influences declarative memory by acting on the encoding, recognition, or both steps of the process, as well as which exact role parietal areas play in declarative memory recognition. The present study aims to disentangle these issues by applying bilateral tDCS, with anode over the left parietal or temporal cortices during the recognition phase of an old-new recognition paradigm. We expect stimulation applied over the temporal cortex to improve performance in old items recognition, since the enhancement of memory traces re-activation, which is linked to this area’s activity [29], might increase accuracy. Stimulation of the parietal area, instead, may lead to different behavioural outcomes; if this region acts as a mnemonic accumulator, a-tDCS should influence detection of new items, since new rather than old items should benefit more from boosting the response-related decision mechanism, the former being harder to process [30]. Alternatively, if this area acts either as an output episodic buffer or as a memory orienting mechanism, we expect that parietal stimulation will modulate performance in old item recognition (similarly to the temporal cortex stimulation), since the activation of memory traces is central in these two latter hypotheses.

## Materials and Methods

### Participants

Forty-nine participants were initially recruited for the experiment; five of these had to be excluded from the analysis: two participants performed below the chance level in the syllable counting part of the encoding task, two had a performance 2 SD below the average performance and thus were considered outliers, and one participant received only 8 minutes of stimulation due to a failure of the stimulator. All remaining participants were right handed, native German speakers, and they had no history of neurological or psychiatric disorders, nor drug or alcohol abuse. None of the participants had metal implants to the head or neck area, pacemaker or deep brain stimulator.

### Ethics statement

The experiment was conducted in accordance with the Declaration of Helsinki and approved by the local ethical committee of the University Medical Center, Göttingen (study number 12/4/12). Written informed consent was obtained from all participants.

### Study design

The present study applied a single-blinded parallel group design with three independent experimental groups for sham, parietal and temporal stimulation conditions. Each participant took part in only one stimulation condition, such that parietal and temporal stimulation group consisted of 15 and sham group of 14 participants. Groups were matched for years of education (*p* = .654), handedness index (*p* = .696) [31], gender (*p* = .76) and age (*p* = 0.352); for descriptive statistics see Table 1).

**Table 1:** The demographic data of the participants in the three independent study groups.

	Age	Years of Education	Laterality Index
**Sham**	**24.80 ± 3.61**	**16.73 ± 2.91**	**92.83± 22.81**
**Parietal**	**23.53 ± 2.61**	**15.90 ± 1.65**	**93.75 ± 11.18**
**Temporal**	**23.13± 3.46**	**16.13 ± 2.87**	**97.33 ± 7.04**

### Material

To assess the declarative memory performance of the participants, a word-list learning task was used. Two lists of words were created, each containing 96 German words, 48 nouns and 48 verbs; the lists were taken from [32] on the basis of ratings from 62 participants included in their study. Wilcoxon signed-rank tests confirmed that the lists were balanced for frequency (computed as the natural logarithm of word frequency to correct the skewedness of the distribution), word length (number of letters, number of syllables), judged familiarity and concreteness, (see Table 2). In addition, seven dummy words were used at the beginning and at the end of the word list encoding and recognition procedure to control for the primacy and the recency effect, respectively. The dummy words were excluded from the analysis. List 1 was always used as study list (i.e., the list to be encoded), while list 2 as control list (i.e., during recognition in combination with list 1).

**Table 2:** The two lists were balanced on the most important psycholingusitic parameters, including the natural logarithm of word frequency, familiarity, letter and syllable length and word concreteness (according to the Wilcoxon signed-rank test).

	List 1 (mean, SD)	List 2 (mean, SD)	Wilcoxon test
**Word frequency**	**1.37 ± 0.77**	**1.39 ± 0.65**	**p = .94**
**Familiarity**	**4.38 ± 0.94**	**4.45 ± 0.92**	**p = .79**
**Letter length**	**7.39 ± 1.5**	**7.42 ± 1.3**	**p = .97**
**Syllable length**	**2.38 ± 0.6**	**2.47 ± 0.63**	**p = .26**
**Concreteness**	**0.63 ± 1.0**	**0.59 ± 0.99**	**p = .74**

### Experimental task

The experimental procedure consisted of an encoding and a recognition phase. During the encoding phase, participants were sequentially presented with items from the study word list. For each of the presented words they were explicitly instructed to first, indicate how many syllables the word contained by button press, and second, to memorise the word for subsequent recognition.

**Fig 1:**
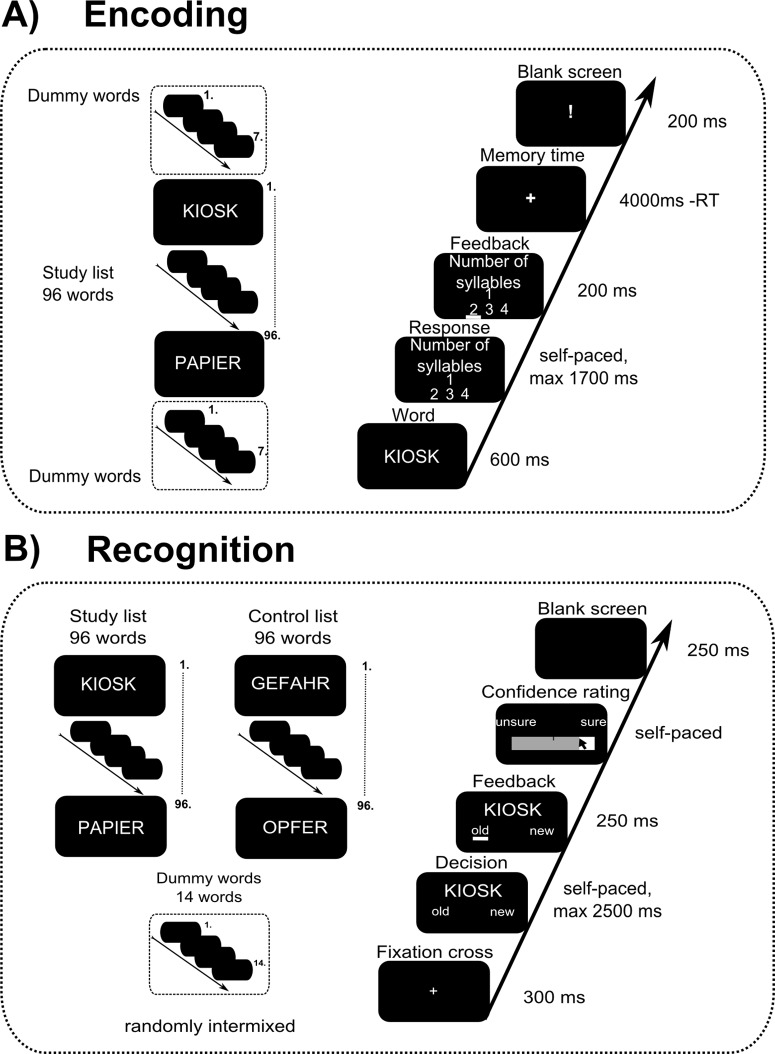
The experimental procedure for the encoding A) and for the recognition B) phase of the verbal learning task.

Fig. 1A illustrates the experimental procedure of the encoding phase. After the presentation of a given word for 600 ms, participants had a maximum of 1700 ms for indicating the number of syllables. Participants’ answers triggered a short highlight about the participant’s decision (irrespective of whether or not it was correct) for 200 ms, followed by a fixation cross in the middle of the screen lasting 4000 ms minus reaction time (RT). This was the time interval dedicated for word encoding. The fixation cross was followed by an exclamation mark, presented for 200 ms, indicating the beginning of the next trial. Each trial’s duration was thus kept constant (5000 ms). The sequence of presentation of the 96 words was randomised for each participant.

Approximately 15 minutes after the encoding session, participants performed the recognition phase of the task. Here, a total of 206 words were presented (96 belonging to the study list, 96 to the control list, and 14 dummy words) one at a time at the centre of the screen. Participants were required to judge whether they had seen the word during the encoding phase (coding it as old) or not (coding it as new). All items were presented in random sequence. In each trial, after a fixation cross (300 ms), the target word was shown for a maximum of 2500 ms. Participants’ response triggered a highlight about their decision (250 ms), followed by a horizontally aligned visual rating scale (VRS) on which participants indicated the confidence level of their answer. Subsequently, a blank screen was shown (250 ms), which was followed by a fixation cross (300 ms) indicating the beginning of the next trial. Fig. 1B illustrates the experimental procedure for the recognition part of the task.

### Stimulation protocol

Direct current (DC) was delivered by a CE-certified DC stimulator (neuroConn GmbH, Ilmenau, Germany), while in sham sessions a microprocessor-controlled multi-channel constant current stimulator (DC-STIMULATOR MC, neuroConn GmbH, Ilmenau, Germany) was used. In all sessions, the DC current was delivered through a pair of conductive rubber electrodes (vertically oriented; 5×7 cm) with paste conductive medium (Ten20 conductive EEG paste, Kappamedical, USA) and was applied during the declarative memory recognition phase. The electrodes were positioned according to the international 10/20 EEG system. In the *parietal* group, electrodes were placed bilaterally over the posterior parietal cortex (a-tDCS over P3 and c-tDCS over P4), whereas in the *temporal* group, the electrodes were positioned over the temporal lobe (a-tDCS over T3 and c-tDCS over T4). The stimulation protocol for these groups included a fade in/out phase each lasting for 30 seconds and the stimulation intensity was set at 1.5 mA with a duration of 15 minutes.

During *sham stimulation*, electrodes were placed identically to the parietal group. The sham sessions included two short stimulation blocks. The first one consisted of a fade-in and fade-out phase, and a stimulation phase at 1.5 mA (all lasting for 30 s for a total duration of 90 s). The second block started 8 minutes after the end of the first block and consisted of a 10 s fade in/fade out phase and a 10 s stimulation phase with 0.8 mA (for a total duration of 30s). The application of this second short stimulation block was for the purpose of maintaining the stimulation-induced cutaneous perception in the participants of the sham group, in order to strengthen the illusion of participating in an active stimulation session. The total amount of charge for the sham session was 0.106 coulomb (C), which is below the minimum charge of 0.18 C necessary for inducing excitability changes [12].

### Cognitive assessment of the study groups

To match the three study groups for verbal working memory and executive functions performance, a reading span test and a 2-back task were administered before stimulation. These tests were administered to assess potential differences in the cognitive performance between the three groups.

#### Reading span

To test the verbal working memory performance, the German version of the standard computerized version of the Reading Span Test was used (adapted from [33]). In this task, participants were presented with a series of sentences (ranging from two to six sentences) and they were required first to read each sentence out loud and to memorise the final word of each sentence. The total number of the subsequently recalled words was taken as dependent variable.

The Shapiro-Wilk test indicated normal distribution (all ps ≥ .13), thus, one-way ANOVA was used to compare the average performance of the three study groups (sham = 78.33 ± 11.52%, parietal = 76.83 ± 10.50%, temporal = 76.00 ± 7.61%). There were no group differences in performance (F < 1).

#### 2-back task

To test for differences in executive functions and working memory performance, a 2-back task was used [34]. In this task, participants were shown a series of letters, presented one at a time, and they were asked to indicate whether the letter on the display was the same as or different from the letter presented two trials before.

Missing values and responses with RTs < 200 ms were excluded from the analysis. We took accuracy calculated on target items and reaction time for correct items as dependent variables. According to the Shapiro-Wilk test, both the accuracy and reaction time data showed non-normal distribution (all ps > 0.004), therefore, a Kruskal-Wallis test was used to compare the mean accuracy (sham = .91 ± .15, parietal = .87 ± .19, temporal = .84 ± .14) and the RTs (sham = 762.5 ± 236.32 msec, parietal = 791.5 ± 321.72 msec, temporal = 804.0 ± 224.72 msec) of the three study groups. The results indicated no significant differences (Bonferroni corrected alpha value = .025) neither in accuracy (χ2(2, N = 45) = 4.87, p = .088) nor in reaction time (χ2(2, N = 45) = 0.5, p = .78).

### Assessing the arousal level and the status of the participants

Before performing the recognition task, the arousal level of the participants was assessed by using a 100-point Likert-scale, where 1 indicated *very tired* and 100 indicated *completely awake*. The questionnaires were administered before the experiment. Due to a failure in the data collection process, the record for one participant in the sham group was missing; thus, this analysis is based on 43 participants in total. The Shapiro-Wilk test indicated non-normal distribution (all ps > .02), and therefore, Kruskal-Wallis test was performed to compare the arousal level of the three study groups prior the experiment (sham = 73.57 ± 23.57; parietal = 80.20 ± 13.88; temporal = 77.63 ± 19.30). No statistical difference was observed between the groups (χ2(2, N = 45) = 0.33, p = .85).

### Statistical analysis of recognition performance

The analyses on recognition performance were performed with the statistical program R [35]. A one way ANOVA has been performed on discriminability index (d’), with Stimulation as grouping independent variable (3 levels: sham, parietal and temporal). Mixed effects models [36] were used as the main statistical procedure for the other considered dependent variables: Accuracy, confidence ratings and reaction times (RT). For RTs, only correct answers and RTs > 200 ms were included in the analysis, while for confidence ratings, missing responses were excluded.

Concerning accuracy, we fitted or data to a series of mixed effects logit model, since accuracy has been considered as a categorical variable. In each model, first we tested whether the inclusion of a different range of fixed effects contributed to the model’s goodness-of-fit. As fixed factors, we considered Stimulation (categorical, 3 levels: Sham, Parietal and Temporal), Item type (categorical, 2 levels: Old vs. New) and Word type (categorical, 2 levels: Verb vs. Noun). The inclusion of a fixed main effect or interaction was evaluated on the basis of likelihood ratio tests, with the final model including only effects which significantly increased the model’s goodness-of-fit [37]. As regarding the random effects structure, a by subject random intercept was added. Finally we tested whether including a by-subject random slope for Item and Word type increased the model’s goodness of fit. For the sake of clarity, only the parameters of the final model will be reported. Moreover, an ANOVA was run on the final model, which will be reported together with significance level approximated with the LRT bootstrap method implemented in the “afex” R package (version 0.6–82, [38]). Post hoc interactions have been explored with the “phia” R package (version 0.1–3,[39]). Concerning RTs, we fitted our data to a series of linear mixed effects models, as they were considered as a continuous variable, following the same procedure used for accuracy. We report the parameters of the final, best-fitting model and the ANOVA run on it, together with their significance level based on Satterthwaite’s degrees of freedom approximation in the “lmerTest” R package (version 2.0–6, [40]). Finally, for confidence ratings, the same procedure adopted for RTs was used, but as fix factor also Accuracy (categorical, 2 levels: Correct vs. Incorrect) was tested. Statistics in the refitted models are reported. We reported a summary of the fixed effects of the final best-fitting models for each variable in a separate table.

## Results

### D’ measures

**Fig 2:**
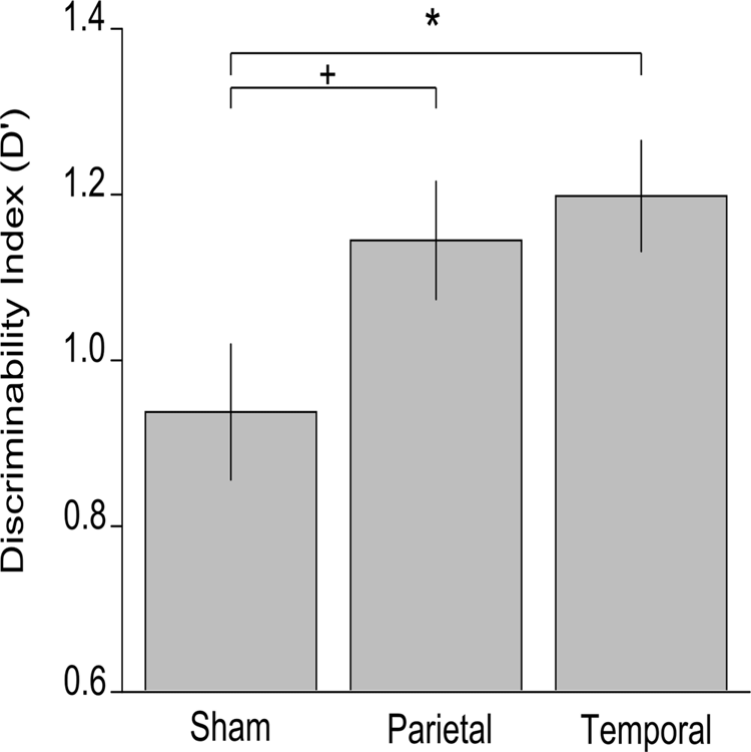
D’ values were significantly and marginally higher for the temporal and parietal groups respectively, compared with the sham stimulation group. D’ value is zero at random choice. Error bars represent standard error of the mean. Asterisk indicates significant differences; the plus sign indicates marginally significant differences.

The mean d’ values for the three simulation groups were the following: .94 ± .32 for the sham group, 1.15 ± .28 for the parietal group, and 1.20 ± .26 for the temporal group (Fig. 2). The ANOVA on d’ measures revealed a significant main effect of Stimulation (*F*(2,41) = 3.363, *p* = .044, ɳ_p_^2^ = .14). Fisher-corrected post-hoc analyses revealed significantly higher d’ values for the temporal (*p* = .018) and marginally significant difference for the parietal (*p* = .057) group with respect to sham group.

### Accuracy measure

**Fig 3:**
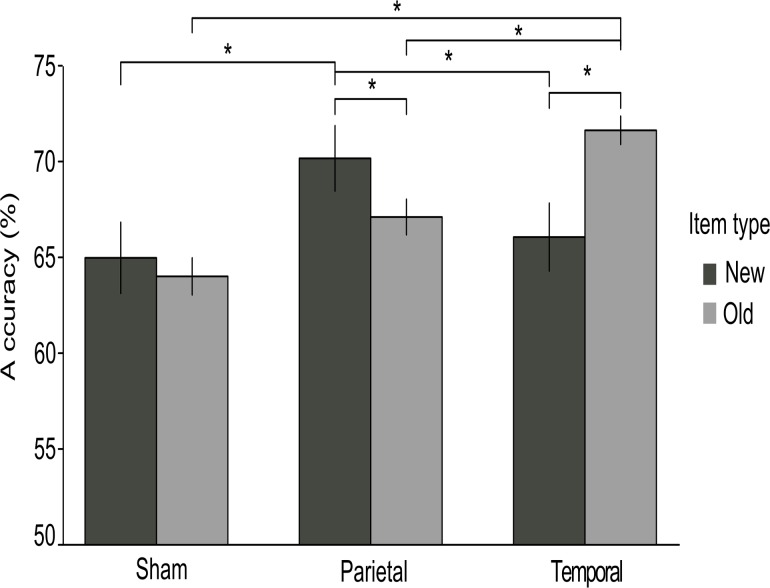
The mean accuracies (%) were significantly higher for both the parietal and the temporal groups compared with the sham stimulation group. Error bars represent standard error of the mean. Asterisks indicate significant differences.

A summary of the fixed effects of the final model run on accuracy is reported in Table 3. Mean accuracy was 67.4 ± 5.3%. Stimulation main effect resulted significant (F(2, 40) = 3.34; *p* = .04), with lower rates for the sham (64.5 ± 5.7%) compared to both the parietal (68.6 ± 5.5%; *p* = .023) and the temporal (68.8 ± 5.9%; *p* = .017) stimulation groups. Critically, the Stimulation by Item type interaction also resulted significant (F(2, 40) = 6.79; *p* = .001). In particular, post-hoc analysis showed that while in the sham stimulation group New and Old words had similar mean accuracy levels (65± 7.2% vs. 64 ± 3.7% respectively; *p* = .51), they differed both in the parietal group, where new items (70.2 ± 6.6%) were better recognized than the old ones (67 ± 3.6%; *p* = .05), and in the temporal stimulation group, where the opposite happened, with old items being better identified than new ones (71.6 ± 2.8% vs. 66 ± 6.8% respectively; *p* = .002). Moreover, accuracy for new items was higher in the parietal group compared to sham (*p* = .018) and temporal (marginally significant, *p* = .054) groups and accuracy for old item was higher in the temporal group compared to the other conditions (sham: *p*<.001; parietal: *p* = .03; see Fig. 3). The main effect of Word type resulted significant (F(1,42) = 68.88; *p*<.0001), since nouns being overall better recognized than verbs (71.6 ± 4% vs. 63.1 ± 4.6% respectively), as well as the Item type by Word type interaction (F(1,42) = 16.88; *p*<.0001). Post hoc analysis on this interaction showed a higher accuracy rate for nouns compared to verbs in both new (73.5 ± 3.1% vs. 60.7 ± 3.8%; *p* = .008) and old items (69.8 ± 3.9% vs. 65.5 ± 4.2%; *p* = .001), but while noun had higher accuracy in new compared to old items (*p*<.001), the opposite was true for verbs, where old verbs were better recognized than new ones (*p* = .002).

**Table 3:** The summary of the fixed effects of the final model taking accuracy as dependent variable (N = 8448; log-likelihood = -5269.6).

Predictor	Coefficient	SE	Z	p
**(Intercept)**	.93	.08	11.40	<.001
**Stimulation Sham: Parietal**	.24	.10	2.40	.018
**Stimulation Sham: Temporal**	.05	.10	.50	.63
**Stimulation Parietal: Temporal**	-.19	.10	-1.90	.05
**Item type New: Old**	-.25	.09	-2.60	.009
**Word type Noun: Verb**	-.58	.07	-8.78	<.001
Stimulation Sham: Parietal * Item type New: Old	-.10	.11	-.91	.36
Stimulation Sham: Temporal * Item type New: Old	.30	.11	2.64	.008
Stimulation Parietal: Temporal * Item type New: Old	.41	.11	3.55	<.001
Item type New: Old * Word type Noun: Verb	.38	.09	4.11	<.001

Note: Random effect for subject intercept had SD of 0.16. SE: standard error of mean.

### Reaction times

A summary of the fixed effects of the final model run on RTs is reported in Table 4. Mean RTs were 1350.9 ± 135 ms for the sham group, 1440.6 ± 173 ms for the parietal and 1341.6 ± 131.3 ms for temporal stimulation group. The final model did not include the fix factor of stimulation or any of its interaction with the other factors. Significant resulted, instead, the main effect of Item type (F(1,5656.6) = 104.85; *p*<.001), with slower reaction times for new (1427.3 ± 159.5 ms) with respect to old (1330.7 ± 173.6 ms) items. Similarly, Word type main effect resulted significant (F(1,5645.9) = 47.01; *p*<.001). Nouns, indeed, yielded faster recognition latencies compared to verbs (1349.1 ± 152.9 vs. 1412.5 ± 160.4 ms respectively).

**Table 4:** The summary of the fixed effects of the final model taking reaction-time as dependent variable (N = 5691; log-likelihood = -39621).

Predictor	Coefficient	SE	df	t	P
**(Intercept)**	1402.36	23.87	50	58.75	<.001
**Item type New: Old**	-100.56	9.82	5657	-10.24	<.001
**Word type Noun: Verb**	66.76	9.74	5646	6.86	<.001

Note: Random effect for subject intercept had SD of 148.7. SE: standard error of mean; df: degrees of freedom.

### Confidence ratings

**Fig 4:**
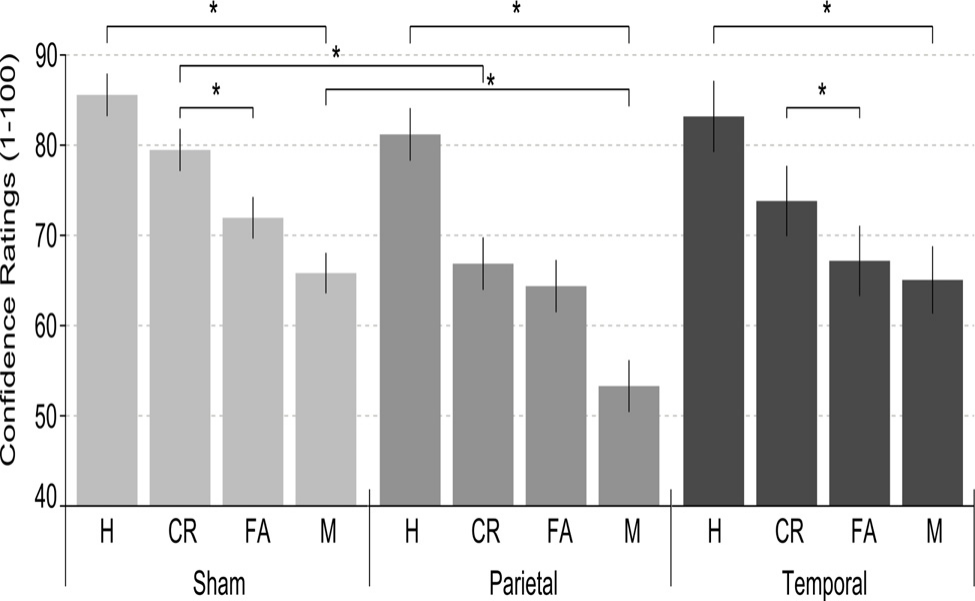
The confidence ratings calculated for hits (H), correct rejections (CR), false alarms (FA) and misses (M) in the sham, parietal and temporal groups, respectively. Error bars represent standard error of the mean. Asterisks indicate significant differences.

A summary of the fixed effects of the final model run on confidence ratings is reported in Tables 5 and 6. Confidence ratings were significantly different for Item type (*F*(1,7738.3) = 4.46; *p* = .035), with old items being associated with higher values than the new ones (76.55% vs. 71.78%). Moreover, accuracy main effect was significant (*F*(1,7738.2) = 512.88; *p*<.001), as correct answers were associated with higher confidence level compared to incorrect ones (78.4% vs. 64.7%). Interestingly, the interaction between Accuracy and Item type was significant (*F*(1,7749.8) = 246; *p*<.001): Hits had a higher confidence level compared to both misses (83.3 vs. 61.5%; *p*<.001) and correct rejections (73.4%; *p*<.001). Conversely, misses had a lower score compared to false alarms (68.1%; *p*<.001). Finally, the Stimulation by Accuracy by Item type interaction resulted significant (F(2,7749.9) = 6.31; *p* = .002). While in the temporal and sham group, both for old and new items, correct answers were associated to higher confidence ratings (all *p*s<.001; see Fig. 4 and Table 6), in the parietal group new correct and incorrect items were associated to similar values (*p* = .31), while old ones differed (*p*<.001). Moreover, correct rejections in the parietal group had lower confidence rating (66.5%) compared to the sham group (78.8%; *p* = .013), as well as misses (54.1% vs. 65.2%; *p* = .029; see Table 6).

**Table 5:** The summary of the fixed effects of the final model taking confidence rating as dependent variable (N = 7787; log-likelihood = 35888).

Predictor	Coefficient	SE	df	t	P
**(Intercept)**	72.86	3.55	48	20.54	<.001
**Stimulation Sham: Parietal**	-7.92	5.14	49	-1.54	.13
**Stimulation Sham: Temporal**	-4.13	4.93	48	-0.84	.41
**Stimulation Parietal: Temporal**	3.79	5.06	49	0.75	.13
**Accuracy 1: 0**	5.98	1.47	7750	4.07	<.001
**Item type New: Old**	-7.66	1.68	7749	-4.57	<.001
Stimulation Sham: Parietal * Accuracy 1: 0	4.37	2.15	7744	-2.03	.04
Stimulation Sham: Temporal * Accuracy 1: 0	-1.70	2.04	7748	-.83	.40
Stimulation Parietal: Temporal * Accuracy 1: 0	2.67	2.12	7743	1.26	.21
Stimulation Sham: Parietal *Item type New: Old	-3.16	2.47	7744	-1.28	.20
Stimulation Sham: Temporal *Item type New: Old	1.29	2.41	7749	.53	.59
Stimulation Parietal: Temporal *Item type New: Old	4.45	2.51	7745	1.77	.08
Accuracy 0: 1 * Item type New: Old	14.64	2.07	7754	7.07	<.001
Stimulation Sham: Parietal * Accuracy 1: 0 * Item type New: Old	10.38	3	7748	3.46	<.001
Stimulation Sham: Temporal * Accuracy 1: 0 * Item type New: Old	2.92	2.93	7754	.99	.32
Stimulation Parietal: Temporal * Accuracy 1: 0 * Item type New: Old	-7.47	3	7748	-2.48	.012

Note: Random effect for subject intercept had SD of 12.49. SE: standard error of mean; df: degrees of freedom.

**Table 6:** Mean confidence level divided by Stimulation group, Item Type and Accuracy.

Stimulation	Item type	Accuracy	Mean Confidency
**Sham**	New	correct	78,84
		incorrect	72,86
	Old	correct	85,82
		incorrect	65,20
**Parietal**	New	correct	66,54
		incorrect	64,93
	Old	correct	80,75
		incorrect	54,12
**Temporal**	New	correct	73,00
		incorrect	68,72
	Old	correct	84,20
		incorrect	62,36

## Discussion

In the present study we investigated by means of tDCS the role of parietal and temporal areas in episodic memory recognition. Critically, recognition memory performance was increased via a-tDCS by stimulating two cortical areas underpinning different aspects of verbal declarative memory. Recognition performance was tested by an old/new decision paradigm, where participants encountered old (learned) and new (unlearned) items in 50–50% of the cases. By using a parallel group design with different participants for sham, parietal and temporal stimulation (matched for demographic and neuro-cognitive status), we found evidence that bilateral stimulation of both parietal and temporal cortices with a-tDCS over the left hemisphere and c-tDCS over the right one effectively boosted recognition memory performance, as evidenced by the increased d’ values for both stimulation groups compared with the sham group. The pattern of improvement found in the current experiment showed an interesting double dissociation between the parietal and temporal groups: While the parietal group reached higher accuracy for new items relative to old ones, the temporal group showed the opposite pattern of improvement.

The higher performance on hits, relative to correct rejections in the temporal stimulation group compared with both sham and parietal stimulation, underlines the importance of this area in memory recollection [29]. In line with a previous study, anodal stimulation of the anterior temporal lobe (ATL) has been proved to effectively modulate episodic memory performance [28]. Uni- and bilateral tDCS over the ATL during encoding and recognition, indeed, decreased false memory rate by 70% percent, without affecting correct memory performance. In contrast to these earlier findings, no difference was found in the false alarm rate between the temporal (0.31) and sham (0.32) groups in the present data. However, there is one critical difference between the two studies that concerns the specific nature of the tasks; while Boggio and colleagues [28] used a test for specifically evoking false memories [41], the present task was not designed for that purpose. On a purely speculative ground, tDCS over the temporal lobes possibly enhanced the recognition of old items, by boosting output information of encoded items in the MTL. This is in line with the trace-reactivation hypothesis proposed by Nyberg and colleagues [29], suggesting that MTL activation during successful recognition reflects the reactivation of the learned information, which might explain why old but not new item recognition was improved by temporal tDCS.

Conversely, the role of the parietal cortex has been linked to different aspects of memory processes including working memory [42] and LTM processes [43,44]—such as acting as a mnemonic accumulator or reflecting the subjective sense of recollection [24,45,46]. Imaging studies on LTM have found greater posterior parietal activations in old/new decision tasks for hits during recognition than correct rejections [43,47,48]. Accordingly, previous evidence highlighted different cognitive processes and cognitive load for correct rejections and hits. For instance, while the recognition of a learned item is based on remember/know process (or both), correct rejections may follow a decision in which the participants take the absence of recollection as evidence for the non-occurrence of the item process also known as “Distinctive heuristic” [49]. Recognition has thus different qualitative characteristics for old and new items. Whereas old items are expected to elicit a vivid recollection or a sense of familiarity as they were previously encoded, new items should elicit no or only an impoverished recollection [50] because no memories of the item are present.

Event-related potential studies, indeed, found different neurophysiological signatures related to hits and correct rejections (as well as for false alarms). For instance, the late positive complex (LPC) component, which is related to responses associated with recollection processes rather than familiarity feeling, and is localised mainly over the left parietal cortex, has been found larger for correct rejections compared with false alarms [51,52]. Since no recollection can arise from those stimuli that were not encoded, and since LPC related to false alarms/correct rejections arise at a later onset compared with the usual LPC elicited by hits, it has been proposed that this activity may reflect left parietal response-related decisional processes [52]. Moreover, an error related negativity (ERN) component recorded at parietal sites has been found larger for false alarms compared with correct rejections; this finding possibly indicates a response competition or a response uncertainty that arises during answer selection in conflict conditions, such as when no memory trace is available and the participant must base their decisions on some criteria [53].

Accordingly, imaging studies support the hypothesis that the parietal cortex may play an important role in the memory decision process [18]; it might act, as a mnemonic accumulator in which memory signals are compared with a decision criteria in order to select whether the presented information had already been encoded or not [21]. In line with these premises, tDCS over the posterior parietal areas might have boosted monitoring and decision processes occurring during our declarative memory task, thus improving novel items detection. It has to be noted that the increase in correct rejection is mirrored, by definition, by a decrease in false alarms. Parietal cortex stimulation might have thus enhanced the decision process by decreasing the rate of false alarms and, in turn, increasing the rate of correct rejections.

An alternative account links parietal cortex activations with the phenomenological experience of recollection [24,45,46]. Similarly, neuropsychological studies found reductions in confidence ratings of patients with uni- and bilateral parietal lesion during recognition/recollection memory processes [54,55]. Although it is difficult to relate these findings to behavioural outcomes of healthy individuals, we expected subjective confidence ratings to be modulated by parietal cortex stimulation applied during the recognition phase. Previous tDCS studies applying stimulation bilaterally over the parietal cortex in episodic memory tasks found contrasting results. The first study applying tDCS during memory encoding reported no modulatory effect of tDCS in confidence ratings [15]. A more recent study in which tDCS was applied during the recognition phase found subtle confidence ratings modulation restrictedly to new items [27]. In a first experiment false alarms and correct rejections (i.e. correct and incorrect decisions on new items) showed similar ratings after parietal stimulation, while in the sham condition false alarm had significantly lower confidence level. In a second experiment correct rejections had higher confidence level in parietal as compared to sham stimulation condition [27]. The pattern of the present results is in accordance with these recent findings in confidence ratings [27]: Correct rejections and false alarms had similar ratings in the parietal while they differed in the sham stimulation group. Nevertheless, these findings are difficult to fit to the perceived oldness hypotheses, as the ratings for the old rather than new items should have been affected by parietal tDCS, or tDCS should have lowered the confidence ratings of incorrect answers (e.g., false alarms or misses) in the parietal group. As suggested in previous studies [27], tDCS may have affected processes related to both true and false recognitions, which have been found to be related to parietal lobe activation [54].

Although it is difficult to relate the effects of tDCS on healthy participants to the neuropsychological literature, it has to be noted that they are based on item recognition confidence ratings, while previous patient studies have found confidence reduction related to the source recollection responses [55,56]. Indeed, bilateral parietal lesion patients are characterized by having impoverished, less vivid episodic memories [57]; we thus speculate that even if parietal cortex might be critical in confidence ratings, further tDCS studies designed to directly assess this process are needed to better understand this relationship.

One of the main limitations of the present study is the lack of control condition for current polarity, as we have applied a-tDCS over the left and c-tDCS over the right hemisphere. Consequently, we did not assess the relative role of the hemispheres and polarity in this current experiment. Since ERP and imaging studies indicated mainly left sided activation patterns for verbal episodic memory retrieval (for an overview about neuroimaging data see: [11]), only right handed participants were included and the anodal stimulation was limited to the left side. However, the results of a previous study indicate that reversing the polarity over the parietal cortex (e.g., left c-tDCS and right a-tDCS) had only a slight influence on the effects of tDCS on memory performance [27]. Finally, as the results of the present experiment are based on 44 participants, one limitation is the relatively low number of participants (i.e., 14/15) in the study groups, which can potentially increase the probability of committing the type I error [58].

In summary, this is the first study systematically investigating the effect of tDCS over the temporal and parietal lobes on recognition memory. Studies so far have investigated the effect of tDCS on declarative memory applied the stimulation either over the prefrontal cortex [14], temporal [28], or parietal cortices but during the encoding phase [15] or during both encoding and recognition [28]. This study further expands on these previous findings by showing that not only is prefrontal stimulation able to modulate episodic memory retrieval [14], but also that the enhancement of excitability of the parietal and temporal cortices during the recognition phase of a memory task can lead to an increased memory performance which possibly indicates a different causal role of these areas in successful memory retrieval.
